# Influence of Solvent Composition on the Performance of Spray-Dried Co-Amorphous Formulations

**DOI:** 10.3390/pharmaceutics10020047

**Published:** 2018-04-12

**Authors:** Jaya Mishra, Thomas Rades, Korbinian Löbmann, Holger Grohganz

**Affiliations:** 1 Department of Pharmacy, University of Copenhagen, Universitetsparken 2, 2100 Copenhagen, Denmark; jaya.mishra@sund.ku.dk (J.M.); thomas.rades@sund.ku.dk (T.R.); Korbinian.loebmann@sund.ku.dk (K.L.); 2 Department of Pharmacy, Faculty of Science and Engineering, Abo Akademi University, 20521 Turku, Finland

**Keywords:** co-amorphous, spray-drying, salt formation, process parameter, stability

## Abstract

Ball-milling is usually used to prepare co-amorphous drug–amino acid (AA) mixtures. In this study, co-amorphous drug–AA mixtures were produced using spray-drying, a scalable industrially preferred preparation method. The influence of the solvent type and solvent composition was investigated. Mixtures of indomethacin (IND) and each of the three AAs arginine, histidine, and lysine were ball-milled and spray-dried at a 1:1 molar ratio, respectively. Spray-drying was performed at different solvent ratios in (a) ethanol and water mixtures and (b) acetone and water mixtures. Different ratios of these solvents were chosen to study the effect of solvent mixtures on co-amorphous formulation. Residual crystallinity, thermal properties, salt/partial salt formation, and powder dissolution profiles of the IND–AA mixtures were investigated and compared to pure crystalline and amorphous IND. It was found that using spray-drying as a preparation method, all IND–AA mixtures could be successfully converted into the respective co-amorphous forms, irrespective of the type of solvent used, but depending on the solvent mixture ratios. Both ball-milled and spray-dried co-amorphous samples showed an enhanced dissolution rate and maintained supersaturation compared to the crystalline and amorphous IND itself. The spray-dried samples resulting in co-amorphous samples were stable for at least seven months of storage.

## 1. Introduction

Oral pharmaceutical products comprise about 70% of all pharmaceutical products, making oral administration the most convenient and preferred route of drug delivery [1,2]. Approximately 40–60% of the currently marketed low-molecular-weight drugs and up to 90% of the current drug development candidates show poor water solubility [3,4]. Using an amorphous form of the drug is an excellent option to improve low aqueous solubility, since both the dissolution rates as well as the apparent solubility are enhanced when converting a crystalline form to its amorphous counterpart [5]. However, a major challenge with the use of amorphous drugs is their low physical stability, that is, their tendency to recrystallize upon formulation, storage, or administration [6]. This has led to an urgent need to increase the understanding of the link between the physicochemical properties of an amorphous drug and potential interactions with common excipients on the overall physical stability of amorphous forms. Polymeric glass solutions are frequently investigated systems in this respect; however, usually a large amount of the polymer is required due to the limited solubility and miscibility of the drug in the polymer. Certain advantages of using low-molecular-weight excipients in the preparation of co-amorphous binary systems with the drug have been confirmed through previous studies, such as a lower amount of excipient, which is required for a stable amorphous system [7,8]. AAs are especially promising excipients as they can form strong molecular interactions with drugs, which may play a crucial role in stabilizing co-amorphous formulations and increasing drug dissolution rates [9,10,11].

The majority of studies on co-amorphous drug–amino acid mixtures used ball-milling (BM) as production technique [11,12,13], which is a difficult process to scale up. In contrast, spray-drying (SD) is a well-established technique in the food, pharmaceutical, and chemical industries and is more easily scaled up to an industrial level [14,15,16]. SD has also been reported as a production technique for polymeric glass solutions [17] and for co-amorphous drug–drug mixtures [18]. Moreover, SD of AAs as a carrier for the pulmonary delivery of biopharmaceuticals also has been reported [19]. However, SD has not yet been extensively used to produce co-amorphous drug–AA mixtures. Recently, co-amorphous drug–AA mixtures of the drug indomethacin (IND) and three basic AAs, arginine (ARG), histidine (HIS), and lysine (LYS), respectively, were produced using SD as the manufacturing technique [9].

IND is a nonsteroidal anti-inflammatory drug (NSAID), classified as a Biopharmaceutics Classification System (BCS) class II drug having low solubility and high permeability. IND has acidic properties and can exist in several polymorphic solid forms and also as an amorphous solid. Since IND (log *p* = 4.05) has hydrophobic properties and the basic AAs (log *p* < −1.5) are hydrophilic, Jensen et al. [9] used binary solvents consisting of the organic solvent acetone and water for SD at fixed solvent ratios. Using spray-drying, IND was successfully converted to an amorphous form with ARG, HIS, and LYS. In contrast, upon ball-milling, only IND–ARG became fully amorphous. The intrinsic dissolution experiments of the co-amorphous spray-dried mixtures showed a nearly 200-fold increase for the spray-dried IND–ARG compared with crystalline or amorphous ball-milled IND. However, no significant increase in intrinsic dissolution was found for SD IND–LYS and SD IND–HIS. Other researchers have discussed that uneven solvent evaporation could occur when using acetone–water mixtures as cosolvents [20]. This may create a risk for drug–AA phase separation leading to a nonhomogeneous SD powder [9]. To further investigate this issue, the current study uses two different organic solvents for SD: ethanol, which is a polar protic solvent; and acetone, which is a polar aprotic solvent. Both these organic solvents were used at different ratios with water. By changing the solvent composition, it is desired to find the applicable formulation parameters under which IND–AA mixtures turn co-amorphous upon spray-drying. In addition to SD, BM was also used in this study as a comparative technique for co-amorphous sample preparation.

## 2. Materials and Methods

### 2.1. Materials

IND was obtained from Fagron (Copenhagen, Denmark) and the 3 basic AAs (ARG, HIS, and LYS) were acquired from Sigma-Aldrich Chemie (Steinheim, Germany). All AAs and the drug used in this study were of reagent grade and used as received. Ethanol and acetone were 99.9% pure (HPLC grade) and received from Sigma Aldrich (Poole, UK). Water (18.2 MΩ), used as the cosolvent in SD, was freshly prepared using a MilliQ water system from LabWater (Los Angeles, CA, USA).

### 2.2. Preparation Methods

#### 2.2.1. Ball-Milling

BM samples were produced as follows: a total mass of 700 mg crystalline IND and AA (ARG, HIS, and LYS, respectively) at a 1:1 molar ratio was introduced to 25 mL milling jars. Two 12 mm stainless steel balls were added. The vibrational ball-mill (MixerMill MM400, Retsch GmbH & Co., Haan, Germany) was placed in a cold room (4 °C). Continuous milling was performed at 30 Hz for up to 60 min.

#### 2.2.2. Spray-Drying

IND and each of the AAs were weighed and combined together at a 1:1 molar ratio, respectively. These mixtures were then dissolved in (i) ethanol and milliQ water; and (ii) acetone and milliQ water, each to give 250 mL of final solutions, respectively. The ethanol and water mixtures contained 95%, 90%, 80%, 60%, 40%, 10%, and 5% (*v*/*v*) ethanol. The acetone and water mixtures contained 85%, 70%, 55%, 45%, 30%, and 15% (*v*/*v*) of acetone. The concentration of IND–AA in the solutions varied from 1.92 mg/mL to 51.4 mg/mL (see Table 1), depending on the aqueous AA solubility and the solubility of IND in the organic solvent. SD was performed using a Büchi B-290 spray-dryer (Büchi Labortechnik AG, Flawil, Switzerland) equipped with an inert loop B-295 (Büchi Labortechnik AG, Flawil, Switzerland). For the ethanol-water samples, SD conditions were as follows: inlet temperature: 140 °C; outlet temperature: 90 °C; atomizing air flow rate: 667 L/h; drying air flow (nitrogen): 40 m^3^/h and feed flow rate: 9 mL/min. For the acetone-water samples, the inlet temperature was decreased to 135 °C and the outlet temperature was 75 °C. The outlet temperature was always maintained below the glass transition temperature of SD IND–AA mixtures.

### 2.3. Analytical Methods

#### 2.3.1. X-ray Powder Diffraction (XRPD)

All drug–AA mixtures prepared by BM and SD were investigated in triplicates by X-ray powder diffraction (XRPD) using an X´Pert PANanalytical PRO X-ray diffractometer (PANanalytical, Almelo, The Netherlands) with Cu kα radiation (1.54187 Å), current: 40 mA and acceleration voltage: 45 kV. Each of the BM and SD samples was scanned at a scan rate of 0.067° 2θ/s and a step size of 0.026° 2θ in the range 2° to 35° 2θ in reflectance mode. The data collected was analysed using X´Pert PANanalytical Collector software (PANanalytical, Almelo, The Netherlands).

#### 2.3.2. Modulated Differential Scanning Calorimetry (mDSC)

Thermal analysis was performed using a Discovery DSC (TA Instruments, New Castle, DE, USA). Each sample weighing approximately 6–8 mg was placed into an aluminium Tzero pan and sealed with an aluminium Tzero lid. Calibration of temperature and enthalpy was carried out with indium. A heating rate of 2 °C/min was used with amplitude of 0.2120 °C and a period of 40 s. Measurements ranged from −20 °C to 180 °C. A constant nitrogen flow rate of 50 mL/min was applied during each measurement. The glass transition temperature (T_g_) was found by analyzing the data collected using Trios software (TA Instruments, New Castle, DE, USA), and reported as the half height of the onset and end set temperature in the reversing heat flow of the thermograms of three independent samples.

#### 2.3.3. Fourier-Transform Infrared Spectroscopy (FTIR)

Infrared spectra were obtained using a Nicolet 380 FTIR (Thermo Scientific, Madison, WI, USA) attached with an attenuated total reflectance accessory (ATR, Smart iTR, Thermo Scientific, Madison, WI, USA). Spectra were collected in triplicates over a range of 4000–400 cm^−1^ (64 scans, resolution 4 cm^−1^). The spectral region from 1500 to 1700 cm^−1^ was investigated for analysis of salt formation with ThermoScientific OMNIC software (version 8.1.11).

#### 2.3.4. Powder Dissolution

A USP type II apparatus (Erweka DT600 dissolution tester, Erweka GmbH, Heusenstamm, Germany) was used to perform powder dissolution of selected co-amorphous formulations. 650 mg samples were added to 100 mL USP 0.1 M phosphate buffer of pH 7.2 as the dissolution medium. Three replicates were conducted for each sample in a modified, custom-made set-up using 250 mL vessels, with rotating minipaddles at 100 rpm and a temperature of 37 °C. Being a down-scaled version of the USP type II apparatus, the minivessel and paddles showed essentially similar hydrodynamics when compared to the standard USP type II apparatus [21]. 1 mL of sample was collected after 10, 30, 60, 120, and 180 min of dissolution testing, using a 0.22-µm latex-free syringe filter (Qmax, Frisinette APS, Knebal, Denmark) and replaced with 1 mL buffer solution. The concentration of IND in the buffer was measured in triplicates by a UV spectrophotometer Evolution 300 (Thermo Scientific, Cambridge, UK) at 264 nm.

### 2.4. Storage

All samples were stored in a desiccator over silica gel (5.4% relative humidity) at room temperature and a physical stability study was performed for all SD samples. A few mg from each sample was taken out and analyzed by XRPD at day 0 and once every month after that. The remaining samples were placed back into the desiccator for further stability studies and the measured samples were discarded.

## 3. Results and Discussion

### 3.1. Preparation of Co-Amorphous Formulations

**Figure 1:**
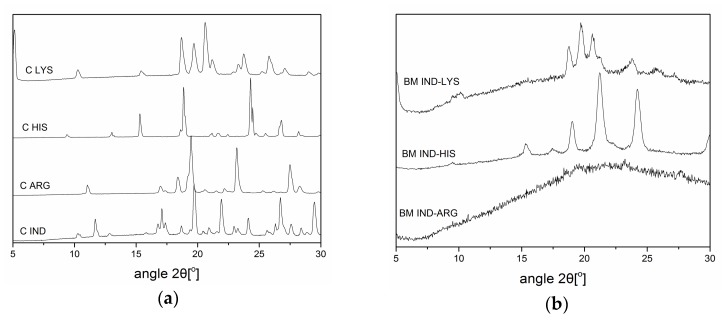
X-ray powder diffraction (XRPD) diffractograms of (**a**) crystalline (C) lysine (LYS), histidine (HIS), arginine (ARG), and indomethacin (IND). (**b**) Mixtures of IND–LYS, IND–HIS, and IND–ARG prepared by ball-milling (BM) for 60 min.

IND–AA mixtures (IND–ARG, IND–HIS, and IND–LYS) at a 1:1 molar ratio were investigated for their ability to form co-amorphous systems using both BM and SD, where the emerging technique of SD was compared to the established technique of BM. BM was performed first to investigate the formation of a co-amorphous system without the use of solvents. Figure 1 shows the XRPD diffractograms of the crystalline raw materials and the mixtures after BM. Only the IND–ARG system was fully amorphous after 60 min of BM, whereas IND–LYS and IND–HIS showed residual crystallinity of the respective amino acids. LYS reflections were observed in BM IND–LYS at 10.1° 2θ, 18.6° 2θ, 19.7° 2θ, 20.6° 2θ, 21.2° 2θ, 23° 2θ, 25° 2θ, and 27° 2θ. On the other hand, IND–HIS showed HIS reflections at 9.4° 2θ, 15° 2θ, 19° 2θ, and 24° 2θ, with some overlapping reflections of HIS/IND (around 21° 2θ) and IND reflections at 17° 2θ and 30° 2θ after 60 min of BM.

Due to this remaining crystallinity, the glass transition temperatures (T_g_s) observed for IND–LYS and IND–HIS do not necessarily represent the systems to be at the 1:1 molar ratio. However, the experimentally determined T_g_s of the BM IND–AAs (110.1 °C, 92.7 °C, and 94.5 °C for IND–ARG, IND–HIS, and IND–LYS, respectively) were applicable to form the basis for the design of the SD process, where the outlet temperature has to be maintained below the T_g_ in order to avoid recrystallization during the process.

**Figure 2:**
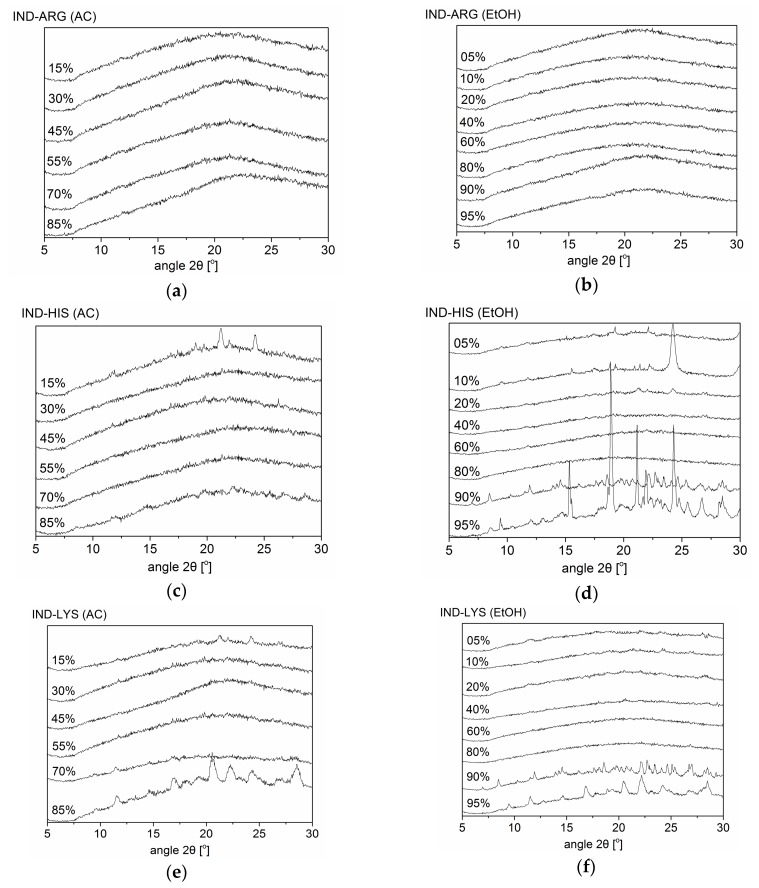
XRPD diffractograms of indomethacin–arginine (IND–ARG), IND–histidine (IND–HIS) and IND–lysine (IND–LYS) mixtures prepared by spray-drying (SD). Organic solvent denoted in percentage (%) is acetone (AC) in (**a**,**c**,**e**); and ethanol (EtOH) in (**b**,**d**,**f**).

Figure 2 shows the XRPD diffractograms of SD IND–AA mixtures at various solvent compositions. SD IND–ARG was found to be co-amorphous for all solvent mixtures used. In contrast to the ball-milling results, amorphous mixtures were also achieved for the majority of solvent mixtures in SD IND–HIS and SD IND–LYS. However, some limitations were observed for these systems upon spray-drying, presumably due to kinetic changes in the solvent composition during processing. Using acetone–water blends, IND–HIS and IND–LYS show crystallization of the respective amino acids HIS and LYS when the acetone concentration is higher than 85% and 70%, respectively, probably due to the low solubility of the AAs in the solvent, due to the high proportion of acetone. Vice versa, systems with an acetone concentration less than 30% show the presence of crystalline IND in SD IND–HIS and SD IND–LYS (Figure 2). This suggests that the drug–AA systems crystallize differently at different organic solvent–water mixture concentrations. Similar findings were seen for the ethanol–water mixtures for SD IND–HIS and SD IND–LYS when the ethanol content was below 20% or above 90%. Thus, both SD IND–HIS and SD IND–LYS require a solvent composition in a certain range to achieve an amorphous product. This means that IND requires a specific minimum concentration of organic solvent (acetone/ethanol), and HIS and LYS require a specific minimum water concentration to form an amorphous product. This makes the solvent fraction an important parameter in co-amorphous formulation, and not just the solvent type (protic/aprotic).

### 3.2. Thermal Analysis of Co-Amorphous Systems

The T_g_ of an amorphous homogeneous glass solution can be calculated theoretically using the Gordon–Taylor (GT) equation (Equation (1)), where T_g1_ and T_g2_ are the glass transition temperatures of the different components in the drug–AA mixtures and *w*_1_ and *w*_2_ are the weight fractions of the drug and AA. Interactions between two components in a binary mixture are not considered in the GT equation.

T_g_ = (w_1_ · T_g1_ + K w_2_ · T_g2_)/(w_1_ + Kw_2_) (1)

The constant K is calculated by Equation (2):

K = (T_g1_ · ρ_1_)/(T_g2_ · ρ_2_) (2)

where ρ_1_, ρ_2_ are the densities of the compounds [22]. The values were calculated using Table 2.

Table 1 summarizes the results of the XRPD, DSC, and FTIR measurements for the various formulations and solvent compositions. It can be seen from Table 1 and Section 3.1 that upon using different organic solvent concentrations, the resulting IND–AAs show differences in product characteristics, such as the T_g_. This is in line with the finding of Patterson et al. [23] that IND-based products showed differences in characteristics such as T_g_ and dissolution rate when produced by different manufacturing techniques. As shown by the XRPD analysis, all investigated SD IND–ARG mixtures became amorphous, irrespective of solvent concentration or the solvent itself. In contrast, SD IND–LYS and SD IND–HIS only became fully amorphous depending on the solvent composition (Figure 2).

**Figure 3:**
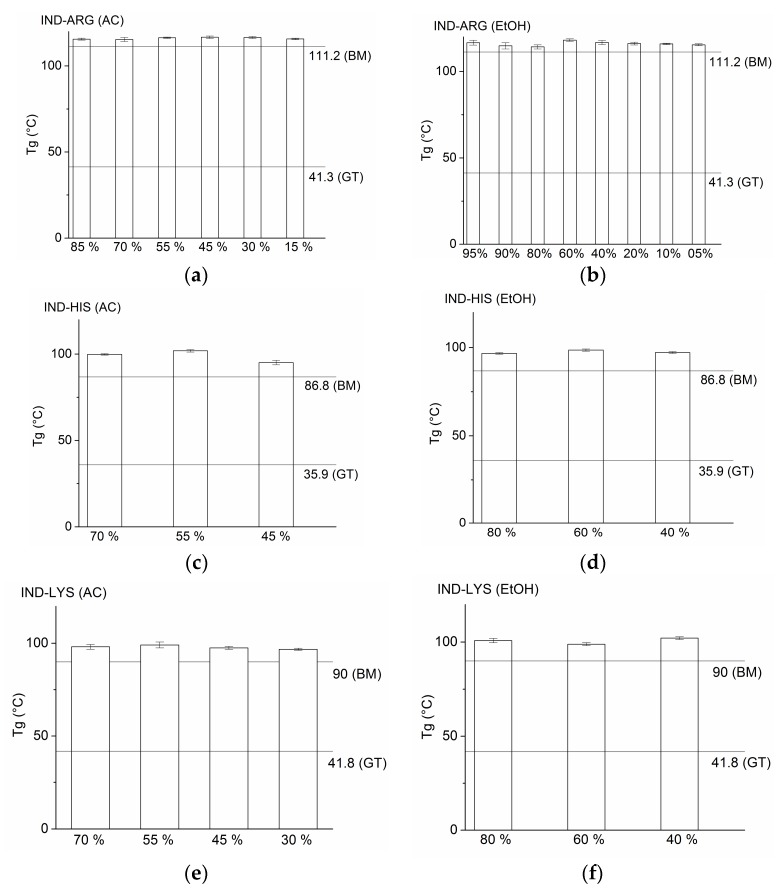
Glass transition temperature (T_g_) of indomethacin–arginine (IND–ARG), IND–histidine (IND–HIS), and IND–lysine (IND–LYS) prepared by different preparation methods. The horizontal lines in each graph indicate the T_g_ measured from ball-milled (BM) samples and theoretical T_g_ calculated using the Gordon–Taylor equation (GT), respectively. Vertical bars represent T_g_ values of spray-dried co-amorphous samples where the organic solvent denoted in percentage (%) is acetone (AC) in (**a**,**c**,**e**); and ethanol (EtOH) in (**b**,**d**,**f**).

The obtained T_g_s of the respective samples in the current study correspond well to the T_g_s obtained in an earlier study by Jensen et al. who found the T_g_s of co-amorphous SD IND–ARG, SD IND–HIS, and SD IND–LYS to be 114.4 °C, 98.0 °C, and 99.5 °C, respectively [9]. The noticeable increase in T_g_ (of around 4–6 °C; see Table 1 and Figure 3) when produced through SD compared to BM (Section 3.1) points to an increased molecular interaction between the drug and AA upon SD. All samples besides IND–HIS spray dried from 30% acetone and IND–LYS spray dried from 20% ethanol show a single T_g_ value. The presence of two T_g_s for the abovementioned systems is attributed to phase separation. In both cases, the second T_g_, which is also the higher T_g_, corresponds to the respective co-amorphous systems. For SD IND–HIS, the first T_g_ is attributed to an IND-rich co-amorphous system, whereas for SD IND–LYS, it corresponds to the T_g_ of the pure IND itself. This confirms that both SD IND–HIS and SD IND–LYS are dependent on the solvent composition and thus require the correct amount of solvent fraction in order to obtain a completely co-amorphous system with a single T_g_. The T_g_ values for all IND–AA mixtures were considerably higher when compared to the T_g_ of the pure drug itself, which was reported to be 36.7 (±0.8) °C [7]. Also, the T_g_s of pure amorphous ARG, HIS, and LYS which were reported as 55 °C, 37.0 (±5.6) °C, and 68 (± 2.1) °C [24,25], respectively, are significantly lower than the T_g_s of the co-amorphous systems reported in this study. Thus, all mixtures show a strong positive deviation from the calculated T_g_ according to the Gordon–Taylor equation, indicating strong molecular interactions between IND and the amino acids. This is presumably due to salt formation and will be more closely investigated in the following section.

### 3.3. Investigation of Molecular Interactions by FTIR Spectroscopy

**Figure 4:**
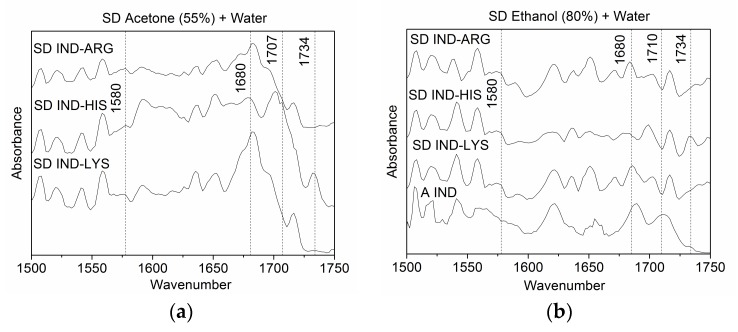
FTIR spectra of co-amorphous indomethacin–arginine (IND–ARG), IND–histidine (HIS), and IND–lysine (LYS) mixtures prepared by spray-drying (SD) with (**a**) 55% acetone as solvent and (**b**) 80% ethanol as solvent. For comparison, the FTIR spectrum of amorphous (A) IND is also shown in (**b**).

Jensen et al. [10] reported FTIR data for the SD IND–AA mixtures used in this study and found complete salt formation only in case of IND–ARG and IND–LYS. Likewise, the influence of the solvent composition on the molecular arrangement for all spray-dried mixtures (mentioned in Table 1) was investigated by the use of FTIR spectroscopy. The region between 1500–1750 cm^−1^ was chosen for analysis as the vibrational modes of the carboxylic acid group, which is responsible for salt formation, lie in this range [8]; the occurrence of salt formation versus partial or no salt formation was found to be independent of the solvent ratio. In this section, two exemplary solvent compositions which resulted in co-amorphous samples were chosen for a detailed investigation: 55% acetone and 80% ethanol. In Figure 4, the FTIR spectra of the co-amorphous mixtures are compared to the FTIR spectra of the amorphous drug itself. Generally, all spectra obtained from co-amorphous mixtures show a strong resemblance with the spectrum of amorphous IND, and it can thus be stated that the spectra are dominated by the vibrations originating from IND. Spectral differences are foremost found in the region 1660–1750 cm^−1^, where the symmetric stretch of the carbonyl group from the free carboxylic acid is located. 

Hydrogen-bonded carboxylic acid vibrations at 1710 cm^−1^ (when spray-dried with acetone–water mixture) and 1707 cm^−1^ (when spray-dried with ethanol–water mixture) and the nonhydrogen bonded acid vibration at 1734 cm^−1^ disappeared in the case of SD IND–ARG and SD IND–LYS compared to the spectra of amorphous IND, due to loss of the proton. This suggests ionisation [8,27] and thus salt formation. In the case of SD IND–HIS, the peak at 1734 cm^−1^ remained present, whereas the peak at 1710 cm^−1^ slightly shifted to 1707 cm^−1^, indicating that no complete salt formation occurred. Thus, the obtained results are in line with earlier findings [9] and confirm salt formation for IND–ARG and IND–LYS. The pKa difference plays an important role in the determination of salt formation as a difference of more than 2 is a preferred scenario for salt formation [28]. In the case of IND and HIS, the pKa difference is only 1.5 and complete salt formation could not be expected, whereas the pKa difference between IND–LYS and IND–ARG is 6.3 and 8.0, suggesting that complete salt formation can be observed.

### 3.4. Dissolution Rate

**Figure 5:**
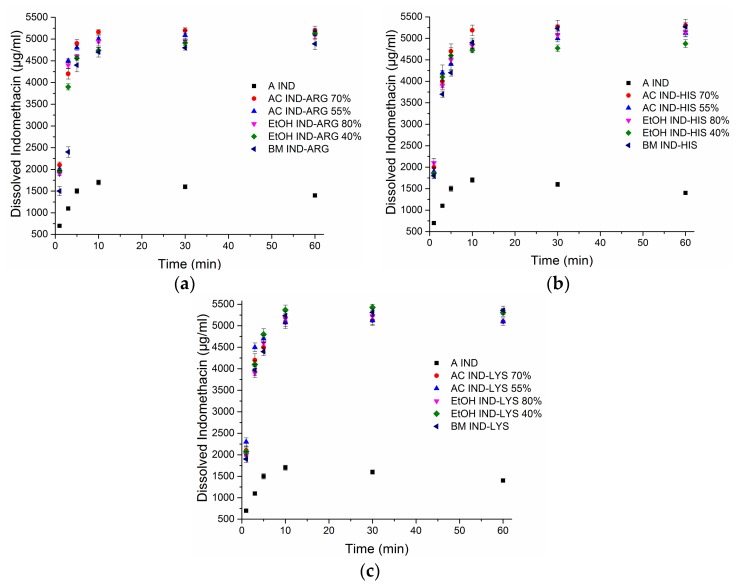
Powder dissolution curves of amorphous (A) indomethacin (IND), spray-dried IND–amino acid mixtures from 70% and 55% acetone (AC) concentration and 80% and 40% ethanol (EtOH) concentration, and ball-milled (BM) IND–amino acid mixtures; for (**a**) arginine (ARG), (**b**) histidine (HIS), and (**c**) lysine (LYS). Saturation solubility of crystalline IND is around 750 µg/mL [26].

An earlier study on spray-dried IND–AA mixtures [10] found the intrinsic dissolution rate (IDR) of SD IND–ARG until 20 min to be much higher than that of both crystalline and amorphous IND. It was also observed that the IDR of SD IND–HIS and SD IND–LYS were similar to each other and significantly lower than that of SD IND–ARG. Based on these earlier findings and the differences in salt formation found in this study, one could expect to see varying results for powder dissolution rates and apparent drug solubility as well. Furthermore, it has been reported that the preparation of amorphous dispersions by different techniques can have significant effects on dissolution rates [29]. The dissolution behaviour of ball-milled and exemplary spray-dried co-amorphous samples of IND–ARG, IND–HIS, and IND–LYS (SD from 70% and 55% acetone concentration, 80% and 40% ethanol concentration) was investigated (Figure 5). The selection of the solvent mixtures was based on the fact that all IND–AA formulations became fully co-amorphous upon SD at these solvent ratios. The dissolution study was carried out at pH 7.2 to ensure complete ionization of IND. The pH of the dissolution media was measured before and at the end of the dissolution experiment and was found to be unchanged.

Irrespective of the amino acid coformer, solvent type, or solvent ratio used, similar release profiles from powder dissolution were observed for all co-amorphous mixtures. Neither could a clear difference be seen between the co-amorphous samples made by the two different preparation methods. For all co-amorphous samples, the maximum dissolved amount is reached within 10 min, followed by a plateau for the measuring duration of 60 min. The pure amorphous IND followed the same pattern, but reached a lower maximum concentration of dissolved drug. Compared to the reported equilibrium solubility of crystalline IND in phosphate buffer at pH 7.2 of around 750 µg/mL reported by Bahl et al. [26], the amorphous IND alone reached a degree of apparent supersaturation (DS) of 2, whilst the co-amorphous mixtures reached a DS of around 4.5 to 5. The high dissolution rate of both BM and SD IND–AA mixtures could be due to the ionization between the drug and the amino acids and the higher water solubility of these amino acids [30]. All the three basic coformers (ARG, HIS, and LYS) showed similar dissolution rates when mixed with the acidic drug IND, irrespective of their different water solubilities (Table 2). Further, the addition of the coformer may prevent recrystallization of the drug, that is, act as a precipitation inhibitor [31].

### 3.5. Physical Stability During Storage

**Figure 6:**
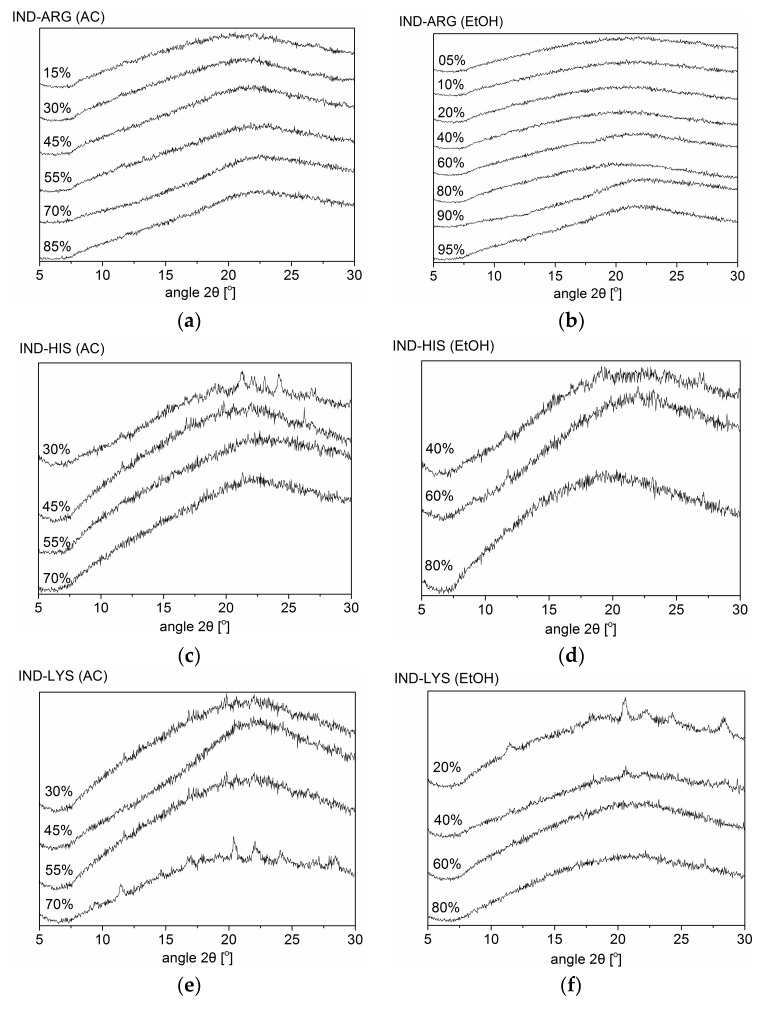
Stability studies (XRPD diffractograms of spray-dried samples) of indomethacin–arginine (IND–ARG), IND–histidine (IND–HIS) and IND–lysine (IND–LYS) mixtures after eight months of storage. Organic solvent denoted in percentage (%) is acetone (AC) in (**a**,**c**,**e**); and ethanol (EtOH) in (**b**,**d**,**f**). (**c**,**e**,**f**) include diffractograms of SD IND–HIS with 30% acetone, SD IND–LYS with 70% acetone, and SD IND–LYS with 20% ethanol, respectively. These samples were co-amorphous until storage month seven.

All samples were analyzed by XRPD at regular time intervals in order to determine their physical stability. Generally, all formed co-amorphous systems were stable for at least eight months of storage, with few exceptions. As could be expected, all IND–ARG systems remained stable throughout the study period. Recrystallization of IND was observed in the XRPD diffractograms for SD IND–HIS and SD IND–LYS spray-dried with 30% and 70% acetone concentrations, respectively; and SD IND–LYS spray-dried with 20% ethanol concentration (Figure 6). It can be seen from Table 1 that these formulations were produced under conditions which were on the boundary of solvent compositions leading to a crystalline product. As ethanol seems to yield stable co-amorphous systems for a larger span of solvent ratios and formulations, it may be considered as a slightly better organic solvent for co-amorphous preparation than acetone.

## 4. Conclusions

This study shows that, in contrast to ball-milling, IND mixtures could be successfully converted into co-amorphous forms by spray-drying with the three amino acids used. The solvent ratio (organic solvent–water) was found to be an important parameter to achieve a co-amorphous formulation. Co-amorphous products could be obtained from both acetone and ethanol water mixtures. IND–ARG always showed a single T_g_ when spray-dried from organic solvent–water mixtures, irrespective of solvent type. For IND–LYS and IND–HIS, a single T_g_ was only observed at intermediate solvent mixture ratios (30% to 80% organic solvent), while extreme ratios led to products with either crystallised amino acid or drug. This suggests that phase separation or crystallization can be prevented by spray-drying IND–AAs at certain solvent mixture fractions. Salt formation was confirmed for both IND–ARG and IND–LYS by FTIR spectroscopy. All BM and SD IND–AAs showed a strong increase in dissolution rate compared to the individual crystalline and amorphous drug, including SD IND–HIS, although no or only partial salt formation was observed in this case. All co-amorphous BM and SD samples remained supersaturated for up to 60 min. Most of the SD co-amorphous IND–AA mixtures were found to be stable for more than eight months at room temperature. It can be concluded from this study that all IND–AA mixtures could be transformed to a co-amorphous form using spray-drying, depending on the correct choice of the type and the amount of organic solvent.

## Figures and Tables

**Table 1:** Characterization of indomethacin–amino acid (IND–AA) mixtures prepared by spray-drying.

IND–AA Mixtures	Solvent Mixture	ORGANIC Solvent Content %:Water Content % (*v*/*v*)	Concentration (mg/mL) in the Spray-Drying Solution	XRPD	T_g_ by mDSC (°C)	T_g_ by GT (°C)	FTIR
IND–ARG	Acetone + water	85:15	51.4	A	115.6 ± 0.6	41.3	SF
		70:30	38.7	A	115.4 ± 1.2	41.3	SF
		55:45	14	A	116.4 ± 0.4	41.3	SF
		45:55	9	A	116.7 ± 0.7	41.3	SF
		30:70	7	A	116.5 ± 0.6	41.3	SF
		15:85	4.8	A	115.7 ± 0.5	41.3	SF
IND–HIS	Acetone + water	85:15	9	C	-	-	PoNSF
		70:30	12	A	99.8 ± 0.4	35.9	PoNSF
		55:45	14.8	A	101.9 ± 0.7	35.9	PoNSF
		45:55	18	A	95.1 ± 1.3	35.9	PoNSF
		30:70	21	A	D: 49.6 ± 1.4, 110.8 ± 0.4	-	PoNSF
		15:85	25	C	-	-	PoNSF
IND–LYS	Acetone + water	85:15	1.92	C	-	-	SF
		70:30	12	A	98.1 ± 1.3	41.8	SF
		55:45	20	A	99.1 ± 1.6	41.8	SF
		45:55	25	A	97.5 ± 0.8	41.8	SF
		30:70	29	A	96.8 ± 0.6	41.8	SF
		15:85	36	C	-	41.8	SF
IND–ARG	Ethanol + water	95:05	15	A	116.6 ± 1.4	41.3	SF
		90:10	12	A	114.8 ± 1.8	41.3	SF
		80:20	9	A	114.2 ± 1.2	41.3	SF
		60:40	7	A	118.1 ± 0.7	41.3	SF
		40:60	5	A	116.7 ± 1.2	41.3	SF
		20:80	3	A	116.1 ± 0.8	41.3	SF
		10:90	2	A	115.9 ± 0.4	41.3	SF
		05:95	1.2	A	115.4 ± 0.6	41.3	SF
IND–HIS	Ethanol + water	95:05	13.5	C	-	-	PoNSF
		90:10	13	C	-	-	PoNSF
		80:20	14.5	A	96.7 ± 0.4	35.9	PoNSF
		60:40	15	A	98.6 ± 0.6	35.9	PoNSF
		40:60	17.5	A	97.8± 0.5	35.9	PoNSF
		20:80	19	C	-	-	PoNSF
		10:90	20.5	C	-	-	PoNSF
		05:95	21	C	-	-	PoNSF
IND–LYS	Ethanol + water	95:05	1.5	C	-	-	SF
		90:10	2.8	C	-	-	SF
		80:20	7	A	100.8 ± 1.2	41.8	SF
		60:40	8.5	A	98.9 ± 0.8	41.8	SF
		40:60	10	A	102.1 ± 0.7	41.8	SF
		20:80	11.2	A	D: 39.6 ± 0.4, 97.5 ± 1.2	-	SF
		10:90	11.8	C	-	-	SF
		05:95	13	C	-	-	SF

mDSC: modulated differential scanning calorimetry, GT: Gordon–Taylor equation, FTIR: Fourier-transform infrared spectroscopy, ARG: Arginine, HIS: Histidine, LYS: Lysine, A: Amorphous, C: Crystalline, D: Double (two) T_g_s, SF: salt formation, PoNSF: partial or no salt formation.

**Table 2:** Density, molar mass, solubility, and pKa of the drug and AAs used in this study [9]. ^a^ Solubility of indomethacin based on phosphate buffer pH 7.2 [26].

Chemical Substances	Density (g/cm^3^)	Molar Mass (g/mol)	Water Solubility (mg/mL)	pKa
Indomethacin	1.379	357.79	0.75 ^a^	4.5
Arginine	1.325	174.2	50	12.48
Histidine	1.412	155.15	41.6	6.04
Lysine	1.237	146.2	100	10.79
